# Ovariotomy*Read before the New York State Veterinary Medical Society.

**Published:** 1894-03

**Authors:** S. E. Willyoung

**Affiliations:** Buffalo. N. Y.


### OVARIOTOMY.*

BY S. E. WILLYOUNG.

This subject is important, owing to its unfrequent appearance in our veterinary literature of to-day, and its unfamiliarity with the general practitioner. The subject is a wide one, and I will not attempt to enlighten anyone on new methods of operating, but will try to demonstrate, *comparatively*, the operation as it is performed by the veterinarian and operators of human surgery.

In 1823 Blundell (Eng.) stated the belief that the *ovaries* could be removed to remedy dysmenorrhœa. After that the subject lay dormant for years, until 1872, when *Hager* of Freeburg, Tait of Birmingham, and Dr. Robt. Battey of Rome, Georgia, each performed the operation. Battey reported his successful case in the Atlanta *Medical and Surgical Journal*, and to him is due the credit of demonstrating to the profession the practicability and value of an operation so universally admitted an honored place in surgery.

Ovariotomy is performed with a two-fold object:

1. Removal of a diseased organ.

2. Abrogation of a physiological function. A complete change of *Life* by *Art*.

---

* Read before the New York State Veterinary Medical Society.

In 1876 Dr. Trenholme of Montreal carried out the operation with a specific cause, his patient being subject to exhausting hemorrhages.

*Operations* in the human being are performed by two methods, *Vaginal* and *Abdominal.* The former now but seldom employed, while the latter is universal.

In the mare, or cow, it can also be done by either vaginal, or flank method. While in the dog the old flank operation is not so practical, as the method of incising the abdomen on the median line, on its inferior surface. While operating after *Battey*, an incision is made in the median line, about three (3) inches in length, just above the pelvis, in the region of the navel. Great care is exercised in preparing the patient physically and mentally, rendering the *parts* and instruments *antiseptic.* The ovaries are, as a rule, ligated or taken off with an *ecraseur.* Hemorrhages are avoided, which always tend to exhaust the patient, while in animals, especially dogs, even a profuse hemorrhage seems to lessen the inflammatory results, and tends to lead to a rapid recovery. According to Battey the peritoneum and abdominal muscles are each sutured separately, usually with animal sutures, and lastly the skin. The whole wound is covered with an antiseptic packing, and bandage, being left on for at least 48 to 60 hours. While in a *dog* the peritoneum, muscle and skin may all be included in one suture, and external applications are necessary in but few cases.

During the past 4 years I have operated on perhaps 200 dogs, among which were collies, spaniels, beagles, pugs, fox terriers, setters, and mongrels. The Scotch collie, I think, is the one best adapted to the operation, and the percentage of recovery is far greater than in other breeds. The mortality I find to be but small, only 3 per cent.

*Age.*—Assuming now we are dealing with healthy female dogs, from 3 to 5 months, and it is always better to operate on one that has never menstruated, as the glands are usually much smaller, and do not play so important a part of the animal. The patient must also be able to inhale and endure an anæsthetic.

*Preparation.*—Dogs should be fed on milk only for two days previous to operating, and care taken the bowels are quite empty. The whole body should be clean, and the hair clipped from the inferior surface of the abdomen.

*Anæsthetics.*—*Squibbs* pure inhalation ether alone should be used, as dogs become anæsthetized more readily, and its after effects are not so lasting. I note but one case where there was vomition after its use. Chloroform produces exhaustion, excites the centre of vomition, and has lasting after effects, which all tend to prevent a rapid recovery.

In the administration of ether, a funnel shaped cone is used, with a piece of cheese cloth stretched across the small end, and a sponge used inside of it. But a few drops of ether should be poured on the sponge at a time, as small quantities often repeated always keep up a more permanent state of anæsthesia.

When the animal is well under the influence of the drug, which is when the muscles of the extremities are relaxed and limp, while an assistant holds the hind limbs apart and elevated, an incision about one and one-half inches between the mammæ, in the median line of the abdomen, just anterior to the pubis, should be made. Incise first the skin, then the muscles, and carefully the peritoneum. Insert a dilator into the opening, shake down the intestines, and the rectum, bladder and uterus are in view. The uterine horns in virgin dogs are easily recognized by their red color, and bifurcating from the anterior portion of the uterus.

The ovaries are found partly attached at the anterior extremity of the horns by cellular, connective and adipose tissue, are small and hard. I usually pick up the horns with the forefinger and thumb, follow them down to the ovary, and excise it with an ordinary pair of small scissors. No notice is paid to the slight hemorrhage that follows, as even though considerable blood be lost they recover rapidly. It is not necessary to absorb the blood, nor to employ antiseptic packing, irrigants, etc., but the whole wound is rapidly sewed up, usually about three stitches are taken which includes the skin and other abdominal walls.

In medical surgery cat gut is generally used, and each part is taken separately, as I stated before, while we use plain silk and include all the tissue in one suture. In the removal of sutures, the veterinarian must be guided by the condition of the wound and the adhesions that exist. As a rule the abdominal muscles have united in from two to three days. and one may be safe in removing the silk. External healing applications are numerous: oxide of zinc, iodoform, boracic acid, pyoktonin or iatrol, are useful. While using pyoktonin on one or two cases, the drug became absorbed to such an extent that the patient lost all appetite, had diarrhœa, became very weak, had diuresis, and the urine assumed a greenish tint. However, recovered rapidly when its use was discarded. Of all applications used, *Iatrol* (I think) gave the most satisfactory results, the wounds generally healing in from six to eight days.

*Results.*—Hernia, peritonitis and septic-infection are a result of ovariotomy, and each may be avoided in a measure by careful operations and proper after-treatment. In case of hernia after the wound has healed, the skin over the sac should be incised, the edges of the muscles scraped, and stitches taken through the skin, muscles and peritoneum, bringing all the parts in close apposition, and treat as before.

*The Effects Physiologically.*—Menstruation is invariably arrested where the entire ovary and fallopian tube is removed. Patients often experience the sensations attendant upon natural menopause, especially vascular and nervous disturbances, as flushing, etc. The vulva of a bitch will flush and swell at regular periods, but never is there any desire expressed.

*Sterility is a certain result.*—In women the sexual feeling and appetite is fortunately not impaired nor the womanly graces lost.

In spaying a female dog we accomplish an aim. Many dogs are useful and attentive after it, as it does not impair their psychological ability, and again we prevent that prolific quality, which tends to populate our streets with howling mongrels and curs.

BUFFALO. N. Y.

